# Isolation of senescent cells by iodixanol (OptiPrep) density gradient‐based separation

**DOI:** 10.1111/cpr.12674

**Published:** 2019-09-13

**Authors:** Kristina Kovacovicova, Manlio Vinciguerra

**Affiliations:** ^1^ Center for Translational Medicine International Clinical Research Center (FNUSA‐ICRC) Brno Czech Republic

## Abstract

**Objectives:**

Chemotherapeutic drugs induce senescence in cancer cells but, unlike replicative senescence or oncogene‐induced senescence, do so rather inefficiently and depending on DNA damage. A thorough understanding of the biology of chemotherapy‐induced senescent cells requires their isolation from a mixed population of adjacent senescent and non‐senescent cancer cells.

**Materials and methods:**

We have developed and optimized a rapid iodixanol (OptiPrep)‐based gradient centrifugation system to identify, isolate and characterize doxorubicin (DXR)‐induced senescent hepatocellular carcinoma (HCC) cells (HepG2 and Huh‐7) in vitro.

**Results:**

After cellular exposure to DXR, we used iodixanol gradient‐based centrifugation to isolate and re‐plate cells on collagen‐coated flasks, despite their low or null proliferative capacity. The isolated cell populations were enriched for DXR‐induced senescent HCC cells, as confirmed by proliferation arrest assay, and β‐galactosidase and DNA damage‐dependent γH2A.X staining.

**Conclusions:**

Analysing pure cultures of chemotherapy‐induced senescent versus non‐responsive cancer cells will increase our knowledge on chemotherapeutic mechanisms of action, and help refine current therapeutic strategies.

## 1 INTRODUCTION

Cellular senescence has fundamental roles in organismal ageing, age‐related diseases, tumourigenesis and tissue regeneration.^1^, ^2^, ^3^, ^4^ There are three major types of cellular senescence: (a) replicative senescence, (b) oncogene‐induced senescence and (c) DNA damage‐induced senescence. Replicative and oncogene‐induced senescence is accompanied by the epigenetic ageing of primary cells while senescence induced by DNA damage on primary or tumour cells (such as that induced by chemotherapeutics during cancer treatment) is not.^5^

Senescent cells are usually studied in their physiological context, that is, adjacent to non‐senescent cells, by imaging and biochemical approaches. However, for both basic and applied research purposes, such as cancer treatment with chemotherapy, it is fundamental to isolate senescent cells from their native context and to culture them to study their properties. Accurate protocols to isolate small numbers of senescent cells from body fluids for downstream analyses, have been recently developed that rely on cell size^6^ or on specific senescent‐cell markers.^7^ These markers refer to the typical phenotypic traits of senescent cells, including: (a) permanent cell‐cycle arrest; (b) persistent DNA damage response; (c) senescence‐associated heterochromatic foci and other epigenetic changes; (d) senescence‐associated secretory phenotype; and (e) altered metabolism, including increased lysosomal and proteosomal activity.^7^ These features are of interest because they can be exploited for selective killing or clearing of senescent cells using a heterogeneous class of drugs known as senolytics.^8^, ^9^

Attempts to isolate senescent cells using surface markers (DcR2, DPP4, oxidized vimentin)^10^, ^11^, ^12^ or fluorescent ubiquitination‐based cell‐cycle indicator technology^13^ have shown promise. However, simple methods to isolate and culture senescent cells—despite their low or null proliferative capacity—are lacking, particularly in the context of chemotherapy‐induced senescence for cancer treatment. Hepatocellular carcinoma (HCC) is a leading cause of cancer‐related death. An increased number of senescent cells are associated with age‐related tissue degeneration towards HCC, and with chemotherapeutic treatment.^9^, ^13^, ^14^, ^15^ Here, we aimed to develop an easy and rapid gradient centrifugation system to identify, isolate and characterize premature senescent HCC cells induced by chemotherapeutic treatment in vitro.

## 2 METHODS

### 2.1 Induction of senescence

Hepatic cell lines (HepG2, Huh‐7) obtained from CLS‐GmbH were cultured in T75 flasks in DMEM (1X) supplemented with 10% foetal bovine serum (FBS), 1% L‐glutamine and antibiotic‐antimycotic solution (ThermoFisher Scientific). At 70% confluence, the cells were exposed to 100 nmol/L doxorubicin (DXR; Sigma‐Aldrich) for 48 hours. The drug was then washed out, and the cells were maintained in complete DMEM for a further 5 days.

### 2.2 Plates/cover glass coating

Cover glasses for microscopy were sterilized by washing in absolute ethanol and placed into 12‐well or 48‐well cell‐culture plates. The cover glasses and plates were briefly washed twice in PBS and then coated with 50 μg/mL bovine collagen coating solution (Cell Applications, San Diego, CA) and incubated at 37°C for 0.5 hour (for plates) or 2 hours (for cover glasses). The coating solution was removed, and the wells were re‐washed twice in PBS. Complete DMEM (containing an antibiotic‐antimycotic solution, 10% FBS, 1% L‐glutamine, 4.5 g/L glucose) was then added before plating the cells.

### 2.3 Gradient‐based separation

Two differently coloured culture media (DMEM with and without phenol red) were used for separation to allow precise gradient preparation. A 60% OptiPrep stock (Axis‐Shield) was diluted to 40% in both media. Different levels of OptiPrep were then prepared as follows: 22%‐25% to separate dead cells, 15% to separate viable small cells and 10% (HepG2) or 5% (Huh‐7) to separate senescent cells. Then, 2 mL 15% OptiPrep was overlaid by 2 mL 10%/ 5% OptiPrep in a falcon tube, and the upper part of the tube was filled with 3 mL complete DMEM without OptiPrep.

To detach the cells, the culture medium was removed from the culture flasks and the cells were washed with PBS (Ca and Mg free) before a 5‐minutes incubation with 2.5 mL TrypLE Express (ThermoFisher Scientific) at 37°C. The cellular suspension in TripLE was diluted to 12 mL in PBS and pelleted by centrifugation for 5 minutes at 250x *g* at room temperature. The pellets were re‐suspended in 1 mL 22%‐25% OptiPrep/DMEM on underlay under the 15% fraction, 10/5% fraction and medium. The prepared separation tube was then centrifuged at 1000x *g* for 30 minutes at room temperature before the 10%/ 5% and medium fractions were transferred to a new tube, diluted again in PBS to 12 mL and pelleted by centrifugation at 300x *g* for 5 minutes at room temperature. Finally, the pellet was re‐suspended in complete culture medium and the cells were seeded onto pre‐coated culture surfaces for 1 hour to allow for the senescent cells to adhere. Upon attachment of the senescent cells, the medium was replaced to eliminate the non‐senescent cells. Control, gradient‐separated DXR‐untreated cells and unseparated DXR‐treated cells were also seeded in parallel onto pre‐coated culture surfaces, and incubated for 24 hours before morphological and functional characterization.

### 2.4 Senescence‐associated (SA) β‐galactosidase staining

Cellular senescence was visualized by X‐gal [5‐bromo‐4‐chloro‐3‐indolyl‐β‐D‐galactopyranoside] (WVR) staining. SA X‐gal‐positive cells were detected as previously described.^16^ For microscopy, the samples were mounted in Mowiol mounting medium (Biotium) and observed under a Pia‐Apochromat 20x 0.8 M27objective on Axio scan Z1 or LSM 7 DUO system (Zeiss). The cells were then semi‐quantitated according to a 0‐to‐5 scoring system, as follows: 0, 0%; 1, 1%‐20%; 2, 21%‐40%; 3, 41%‐60%; 4, 61%‐80% and 5, 81%‐100% positive cells per field.

Cellular senescence was quantified using CF12FDG [5‐Dodecanoylaminofluorescein Di‐β‐DGalactopyranoside] (Satereh Biotech) by flow cytometry.^9^ Following mixtures of cells were prepared: 60:40 (60% of control cells and 40% of DXR‐induced cells), 50:50 (50% of control cells and 50% of DXR‐induced cells) and 30:70 (30% of control cells and 70% of DXR‐induced cells). Those samples were subsequently divided and halve of the initial thought gradient centrifugation. The HepG2 hepatocyte suspensions were analysed using a flow cytometer FACSCanto (BD Biosciences).

### 2.5 Proliferation assay

EdU (5‐ethynyl‐2‐deoxyuridine) supplied with the Click‐iT EdU Alexa Fluor 555 Imaging Kit (Thermo‐Fisher Scientific), was diluted in DMSO to a final concentration of 10 mmol/L and stored at –20°C. EdU was added to HepG2 and Huh‐7 cultures exposed or not to DXR and cultured on collagen pre‐coated 10 mm cover glass in 24‐well plates, to a final concentration of 10 μmol/L for 36 hours at room temperature until collection. The cells were fixed for 15 minutes in 4% paraformaldehyde in PBS, washed three times with 1 mL 3% BSA/PBS and then permeabilized by 0.5% Triton X‐100 (PBS‐Tx) for 20 minutes (all at room temperature). The Click‐iT EdU reaction cocktail was prepared according to manufacturer's instructions and incubated for 30 minutes at room temperature, protected from light. The solution was removed, and the cover glasses were washed with 3% BSA/PBS. DNA was then stained by Hoechst 33342 (final concentration 5 μg/mL), and the slides were mounted in EverBrite™ Mounting Medium (Biotium) before being scanned by an Axio scan Z1 or LSM 7 DUO system (Zeiss).

### 2.6 Immunofluorescence

Immunofluorescence in HCC cells was performed as previously described.^17^ Briefly, cells were fixed in 4% paraformaldehyde for 20 minutes and permeabilized with 0.1% Triton X‐100 (Sigma‐Aldrich) for 15 minutes. Primary antibodies were from Abcam (anti‐gamma H2A.X (phospho S139), anti‐CDKN2A/p16INK4a, anti‐p21 [CP74]; diluted 1:500), Santa Cruz Biotechnology (anti‐p53 (FL‐393); diluted 1:1000) and R&D Systems (anti‐DPPIV/CD26; diluted 1:1000). The staining was developed using Alexa Fluor‐conjugated secondary antibodies (488, 555 or 633, ThermoFisher Scientific). Cover glasses were placed into EverBrite Hardset Mounting Medium with DAPI (Biotium), and images were acquired using an Axio scan Z.1 or LSM 7 DUO system (Zeiss) confocal microscope.

### 2.7 Data analysis

Image analysis was performed using ImageJ (http:// rsb.info.nih.gov/ij/), ZEN 2011 SP1 (black edition) version 8.1., or ZEN 2 version 2.0.0.0. (Carl Zeiss Microscopy GmbH). C_12_FDG SA‐β‐gal positivity was scored using FlowJo (Becton, Dickinson and Company).

### 2.8 Statistical analyses

The data are expressed as the means ± SEM. Comparisons between groups were performed with the non‐parametric Mann‐Whitney U‐test or Fischer´s exact test as appropriate, using GraphPad Prism Software (version 7.05 for Windows). A *P*‐value ⩽.05 was considered statistically significant.

## 3 RESULTS

### 3.1 Iodixanol density gradient‐based centrifugation separates DXR‐induced senescent HCC cells

**Figure 1:**
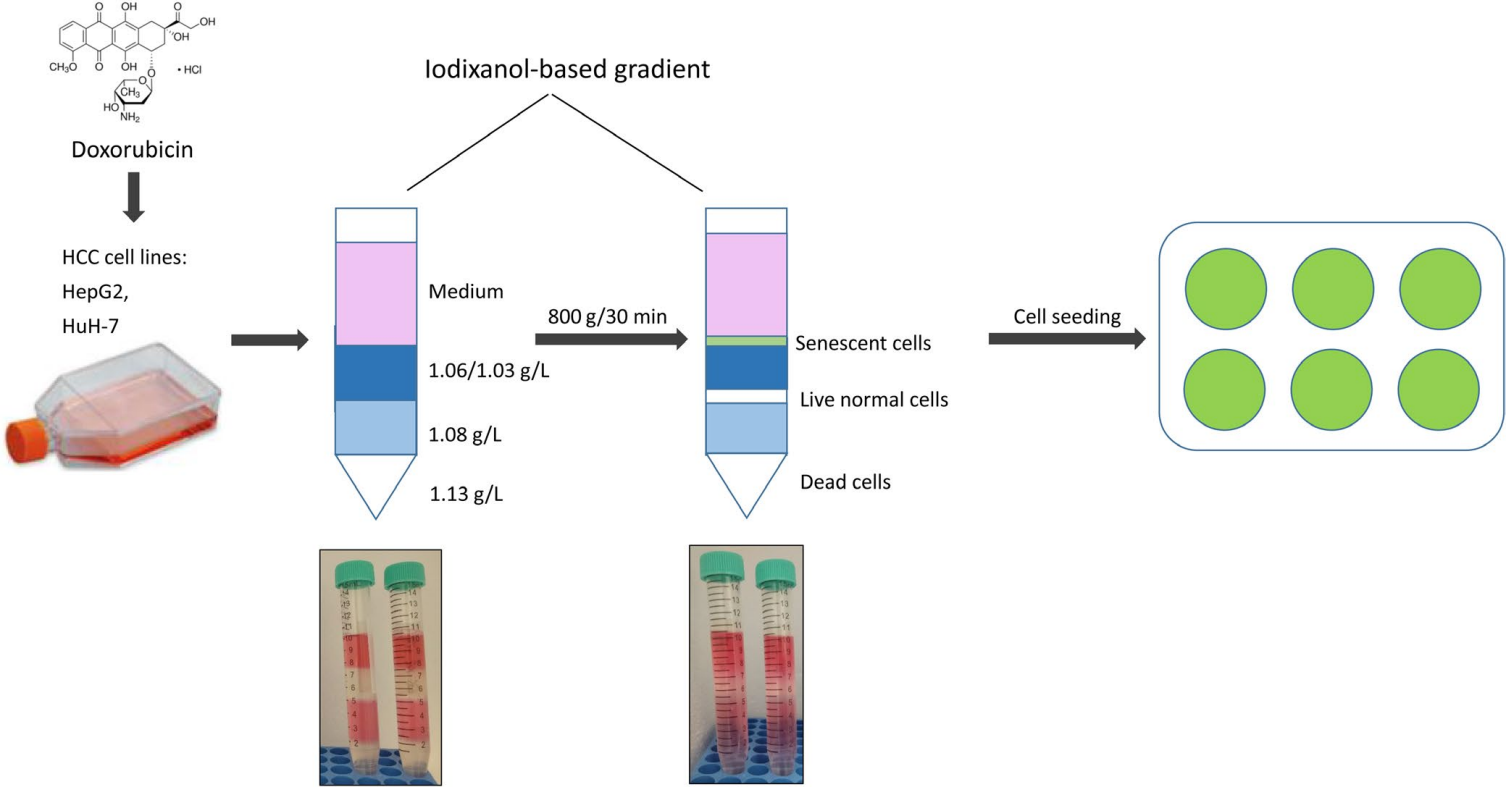
Workflow to isolate and plate doxorubicin‐induced senescent hepatocellular carcinoma (HCC; HepG2, Huh‐7) cells using iodixanol density gradient‐based separation. See text for details

We aimed to develop a procedure to readily isolate and culture HCC senescent cells after exposure to DXR^9^ (Figure 1). The simplest form of separation by centrifugation is differential centrifugation, whereby cells of different densities or sizes in a suspension will sediment at different rates, with the larger and denser cells sedimenting faster. To avoid cross‐contamination, rate‐zonal centrifugation allows for the cell sample to be layered as a narrow zone on top of a density gradient. In this way, the faster sedimenting cells are not contaminated by the slower cells. The gradient stabilizes the cell layers and provides a medium of increasing density and viscosity.

We first prepared a working and gradient solution for cells using OptiPrep^TM^, a 60% (w/v) solution of iodixanol (5,5′‐[(2‐hydroxy‐1‐3 propanediyl)‐bis(acetylamino)]) bis [N,N’‐bis(2,3dihydroxypropyl)‐2,4,6‐triiodo‐1,3‐benzenecarboxamide] in water. We chose iodixanol as it forms more stable and easy‐to‐prepare gradients than sucrose.^18^ We exposed two established HCC cell lines (HepG2, Huh‐7) to 100 nnol/L DXR for 48 hours. This concentration is only slightly cytotoxic to HCC cells, inducing senescence in ~40% of cells.^9^, ^15^, ^19^ We then washed out the DXR and incubated the cells in normal medium for a further 5 days. Upon trypsin‐mediated cell detachment, we deposited unseparated cell pellets on an iodixanol‐based gradient comprising the following layers (from top to bottom): normal cell medium [DMEM, supplemented with 10% foetal bovine serum (FBS), with 1% penicillin/streptomycin], then 1.03/1.06 g/L, 1.08 g/L and 1.13 g/L iodixanol. Over 30 minutes rate‐zonal centrifugation at 800 x*g*, clearly separated (but not uniform) layers appeared, with the senescent cells sedimenting between the cell medium and the 1.03/1.06 g/L iodixanol layer, the live non‐senescent cells sedimenting between the 1.03/1.06 g/L and the 1.08 g/L iodixanol layers, and dead cells sedimenting within the 1.13 g/L iodixanol layer (Figure 1). We then seeded control (CTL, gradient‐separated DXR‐untreated) cells, gradient‐separated DXR‐induced senescent cells (DXR SEP) or unseparated DXR‐treated (DXR UNS, not undergoing gradient‐mediated separation) cells on 24‐well collagen (50 μg/mL) pre‐coated plates for morphological and functional characterization, 24 hours after re‐plating.

### 3.2 Gradient‐based separation enriches for β‐galactosidase‐positive, proliferation‐arrested DXR‐induced senescent HCC cells

**Figure 2:**
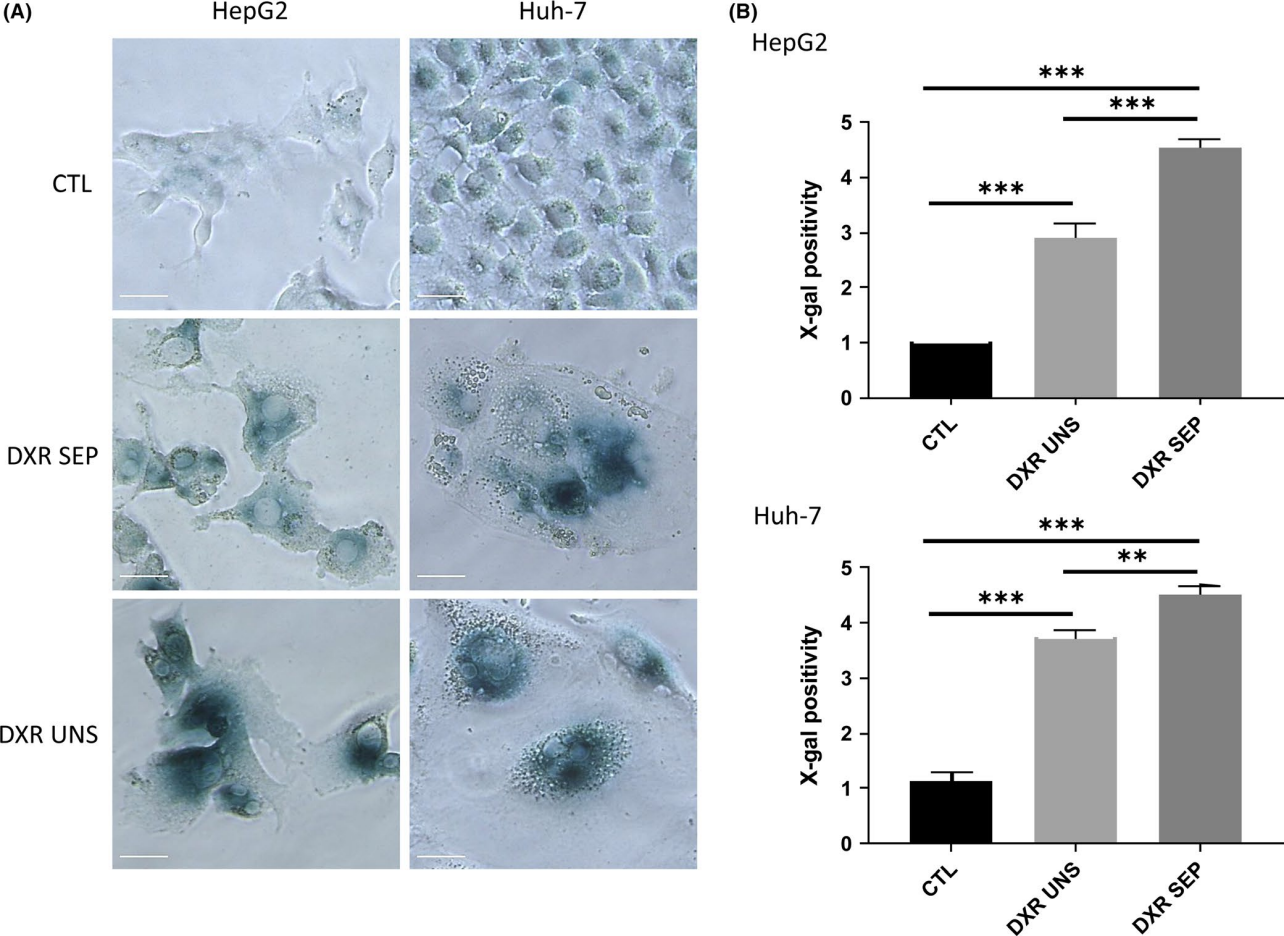
Doxorubicin (DXR)‐induced senescence in HepG2 and Huh‐7 cells treated with 100 nmol/L DXR for 48 h, followed by 5 d washout. DXR‐treated cells were separated (SEP) or not (UNS) by iodixanol density gradient‐based centrifugation, and then re‐plated. Staining was performed 24 h after re‐plating. CTL: gradient‐separated DXR‐untreated control cells. (A and B) X‐gal staining for β‐galactosidase activity in HepG2 and Huh‐7 cells. (A) Images were captured under a bright light microscope for β‐gal. (B) The proportion of positive cells for X‐gal as in (A) was calculated over a total of ~1000 cells per condition. The results are expressed on a semi‐quantitative scale (0, 0%; 1, 1%‐20%; 2, 21%‐40%; 3, 41%‐60%; 4, 61%‐80%; 5, 81%‐100%). Data are expressed as the means ± SEM. n = 6‐7. *P < .05; **P < .01; ***P < .001. CTL, untreated control

To determine the efficacy of the iodixanol density gradient to enrich DXR‐induced senescent HCC cells, we used X‐gal staining to identify the level of SA β‐gal positivity—a standard marker of senescent cells.^20^ HepG2 and Huh‐7 cell lines treated with 100 nmol/L DXR for 48 hours followed by 5 days washout, exhibited a characteristic‐flattened morphology (Figure 2A) compared with rounded untreated cells. Imaging‐assisted semi‐quantitative analyses revealed that ~55% DXR‐treated HepG2 cells and ~70% DXR‐treated Huh‐7 cells were positive for β‐galactosidase prior to gradient‐based separation (Figure 2A,B). After iodixanol density gradient‐based separation and re‐plating, ~85%‐90% DXR‐treated cells were positive for β‐galactosidase—a significant enrichment (*P* < .05) in both cell lines.

**Figure 3:**
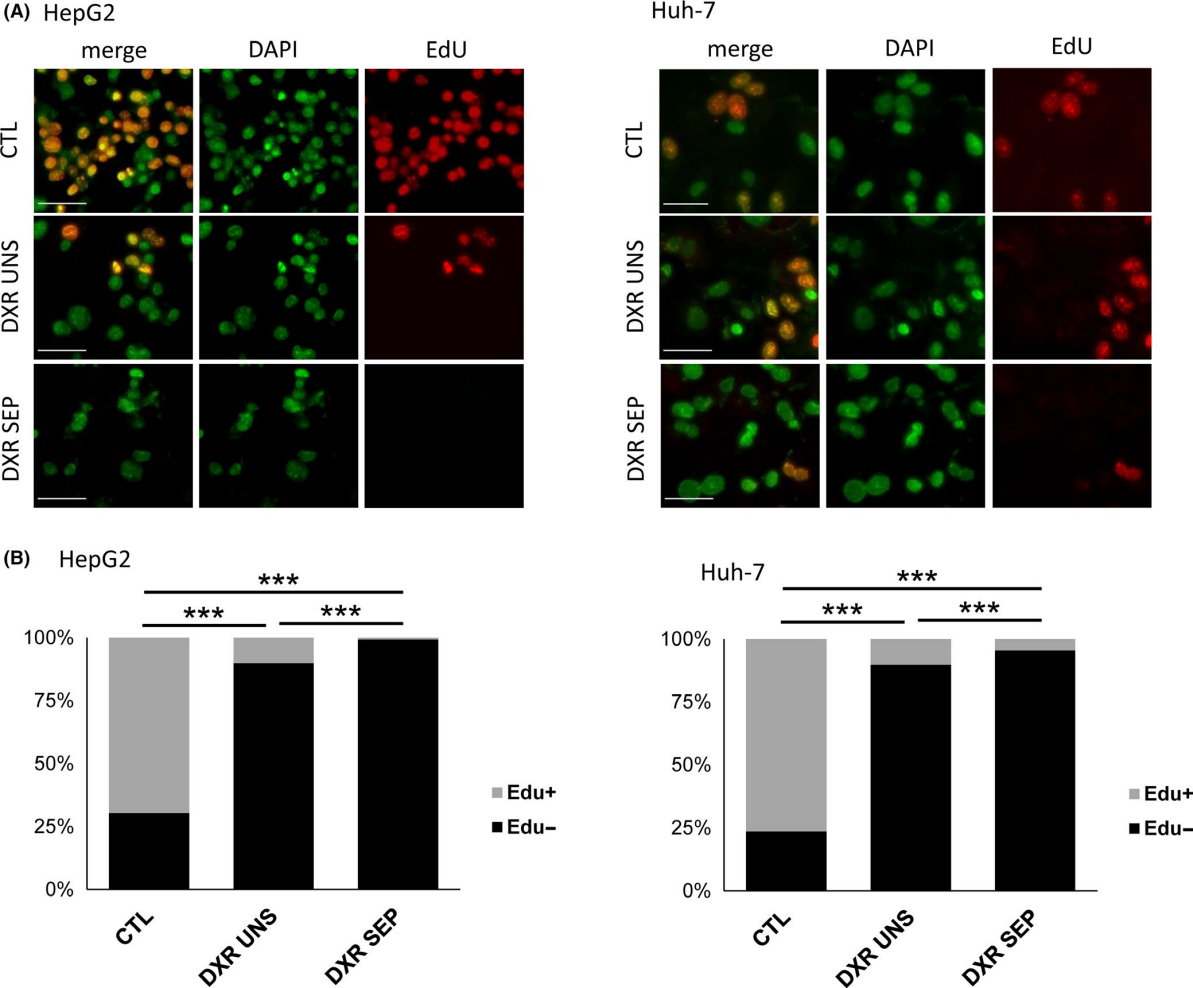
Growth inhibition in HepG2 and Huh‐7 cells treated with 100 nM doxorubicin (DXR) for 48 h followed by 5 d washout. (A and B) EdU staining of HepG2 and Huh‐7 cells. (A) Representative micrographs showing DAPI, EdU and merged labelling in control (CTL) and DXR‐treated cells, separated (SEP) or not (UNS) by gradient‐based centrifugation. (B) Quantification of EdU staining intensity. The proportion of Edu‐positive cells as in (A) was calculated over a total of ~1300 cells per condition. n = 3‐4. **P < .01; ***P < .001

Cell senescence is also referred to as an arrest in cell proliferation. We thus used an immunofluorescence‐based microscopy method based on the cellular incorporation of EdU (5‐ethynyl‐2'‐deoxyuridine), which is a nucleoside analog of thymidine that is incorporated into DNA during active DNA synthesis. In this assay, EdU is fluorescently labelled with a bright, photostable Alexa Fluor® 555 dye. In untreated HepG2 and Huh‐7 cells, 70%‐80% of cells were positive for Edu staining, indicating that the majority of cells were actively proliferating (Figure 3A,B). In cells exposed to 100 nmol/L DXR for 48 hours followed by 5 days washout, the proportion of EdU‐positive proliferating cells reduced to ~10% in both cell lines (Figure 3A,B). After iodixanol density gradient‐based separation and re‐plating, <1% DXR‐treated HepG2 cells and ~3% DXR‐treated Huh‐7 cells remained positive for EdU staining. Gradient‐based depletion of proliferating HCC cells was thus highly significant for both HepG2 and Huh‐7 cell cultures (*P* < .001, Figure 3A,B). Altogether, these data indicate that the iodixanol‐based centrifugation method enriches for senescent HCC cells from a mixed population.

### 3.3 Gradient‐based separation enriches for DXR‐induced DNA damage‐dependent, γH2A.X‐positive HCC senescent cells

**Figure 4:**
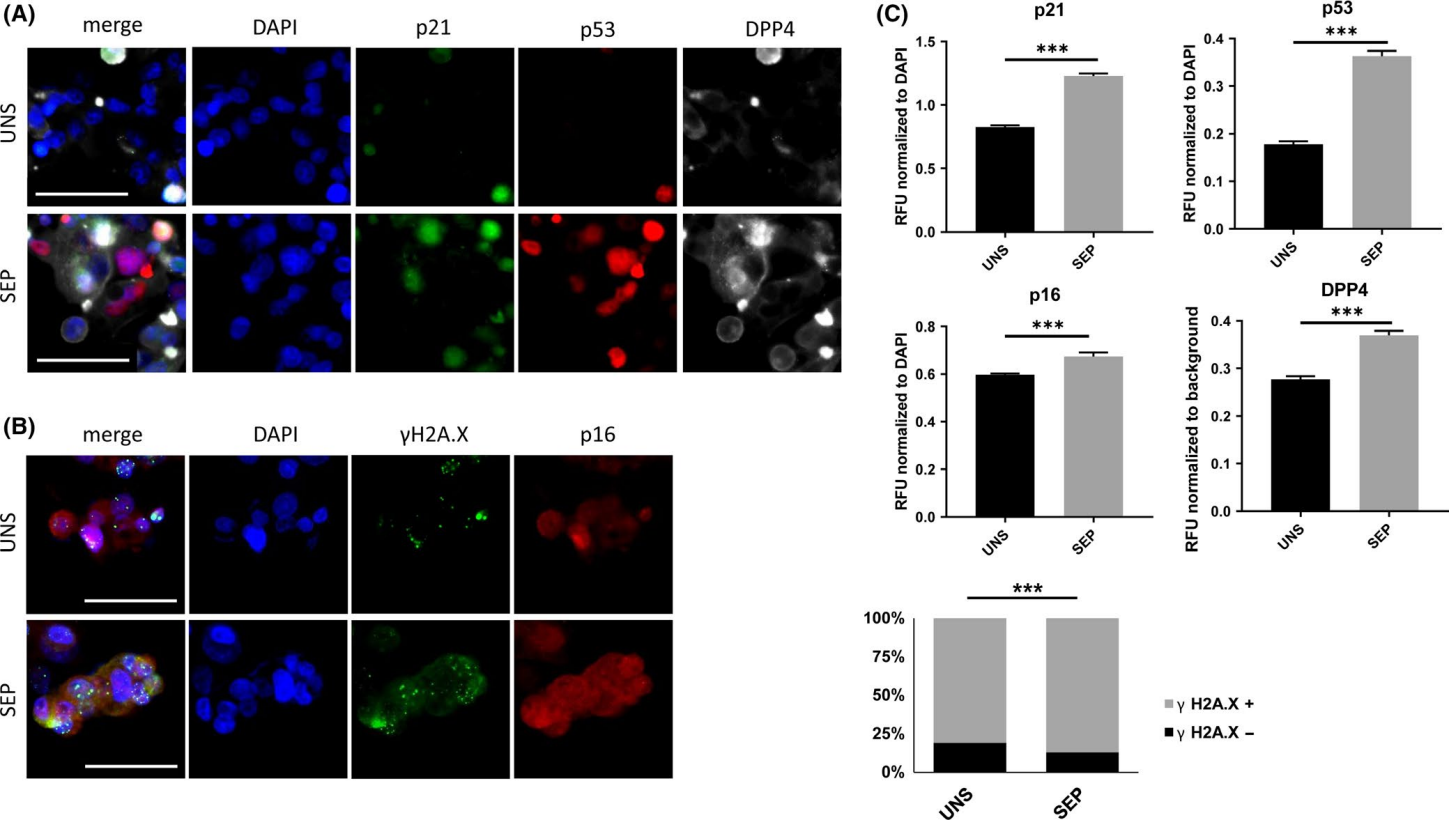
Immunofluorescent detection of selected markers of senescence. HepG2 cells were treated with 100 nmol/L DXR for 48 h, followed by 5 days washout. DXR‐treatment was followed by iodixanol density gradient‐based centrifugation (separated, SEP) or not (unseparated, UNS) and fixed 24 h after re‐plating. (A) Representative micrographs displaying DAPI, p21, p53 and DPP4 staining, and merge, in DXR‐treated, SEP or UNS by gradient‐based centrifugation. Scale bar represents 50 μm. (B) Representative micrographs displaying DAPI, γH2A.X and p16 staining, and merge, in control and DXR‐treated, SEP or UNS by gradient‐based centrifugation. Scale bar represents 50 μm. (C) Normalized quantification of p21, p53, p16 and DPP4 staining intensity and scoring of γH2A.X nuclei, calculated over a total of ~600 cells per condition. n = 4. ***P < .001

**Figure 5:**
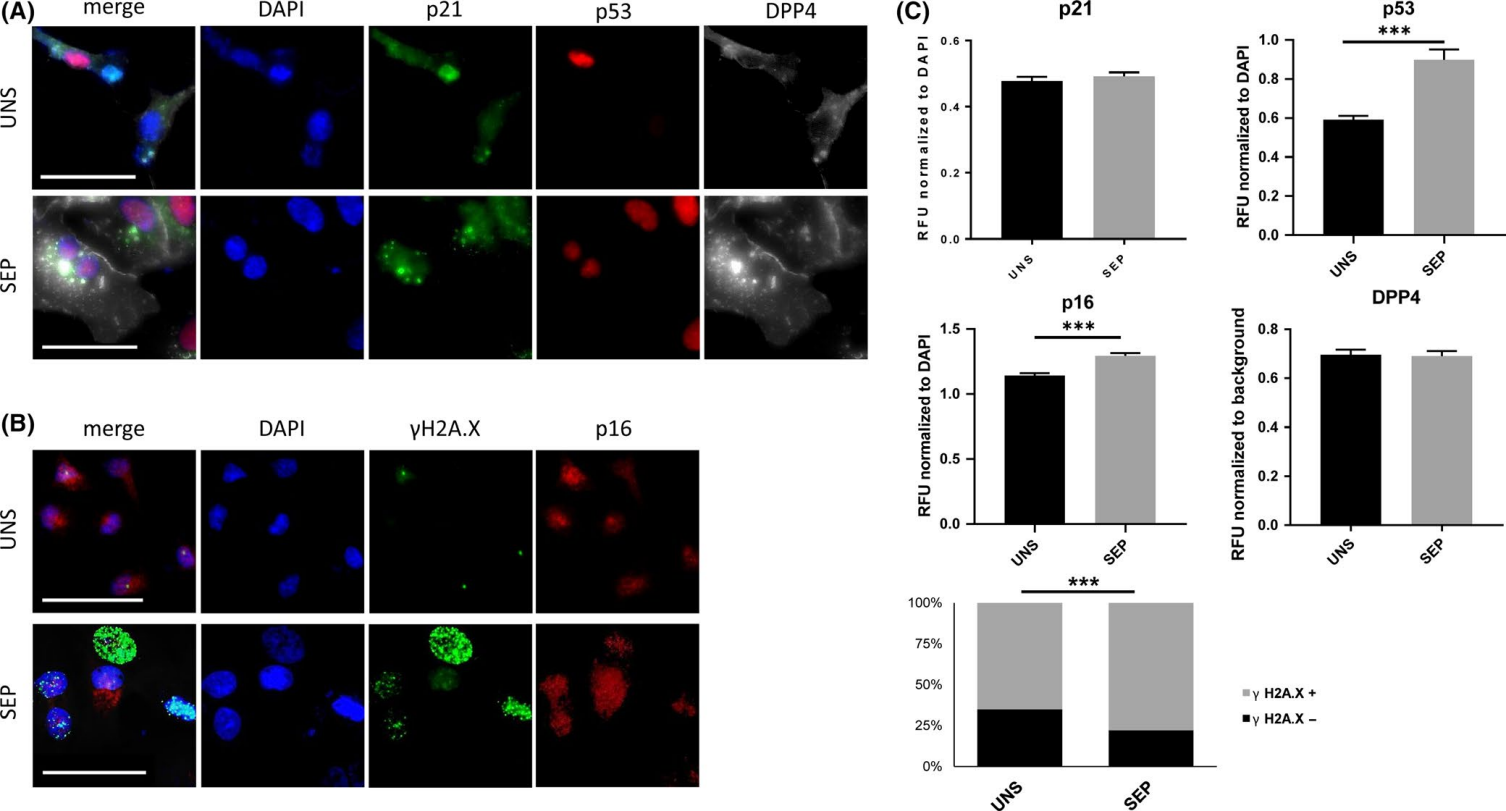
Immunofluorescent detection of selected markers of senescence. Huh‐7 cells were treated with 100 nmol/L DXR for 48 h, followed by 5 d washout. DXR‐treatment was followed by iodixanol density gradient‐based centrifugation (separated, SEP) or not (unseparated, UNS) and fixed 24 h after re‐plating. (A) Representative micrographs displaying DAPI, p21, p53 and DPP4 staining, and merge, in DXR‐treated, SEP or UNS by gradient‐based centrifugation. Scale bar represents 50 μm. (B) Representative micrographs displaying DAPI, γH2A.X and p16 staining, and merge, in control and DXR‐treated, SEP or UNS by gradient‐based centrifugation. Scale bar represents 50 μm. (C) Normalized quantification of p21, p53, p16 and DPP4 staining intensity and scoring of γH2A.X nuclei, calculated over a total of ~ 600 cells per condition. n = 4. ***P < .001

We examined the positivity of DXR‐treated HCC cells for established markers of cell senescence: p21, p53, p16 and DPP4.^7^, ^11^ p16 and p53 nuclear intensity levels were significantly increased in both HepG2 and Huh‐7 cells upon gradient‐based separation (Figures 4 and 5A‐C); HepG2 cells also showed an increase in p21 and DPP4 immuno‐positivity (Figures 4 and 5A‐C). We next quantified the level of γH2A.X activation as an indicator of DNA damage in DXR‐treated HCC cells.^21^ As expected, DXR‐treatment induced a significant increase in the number of distinct γH2A.X foci marking DNA lesions in HepG2 and Huh‐7 cells (Figures 4 and 5B,C). After iodixanol density gradient‐based separation and re‐plating, the number of DXR‐treated cells positive for γH2A.X increased in both HepG2 (from 81% in unseparated cells to 87.4% in separated cells) and Huh‐7 (from 66.5% in unseparated cells to 85.1% in separated cells) (*P* < .001, Figures 4 and 5B,C). These data show that, with some cell type‐dependent differences, an iodixanol gradient‐based separation method significantly enriches the population of DXR‐induced senescent cells, as assessed by the analysis of multiple markers of cell senescence.

Finally, we sought to determine the fidelity of our method. We first induced senescence in HepG2 cells by 48 hours treatment with 100 nmol/L DXR, followed by 5 days washout. The DXR‐induced senescent cells and control (untreated) cells were processed for staining using the fluorogenic substrate of SA‐β‐galactosidase (C12FDG, or FDG) and for flow cytometry analysis, as we previously described.^9^ The following cell mixtures were prepared: (a) 60:40 (60% of control cells and 40% of DXR‐induced cells); (b) 50:50 (50% of control cells and 50% of DXR‐induced cells); (c) 30:70 (30% of control cells and 70% of DXR‐induced cells). The samples were divided into two parts: one part was fixed immediately (UNS) and the other part was processed thought gradient centrifugation (SEP). As shown in Figure S1, irrespective of the starting ratios (60:40, 50:50 or 30:70), we observed a loss of FDG+ cell population upon separation (SEP condition compared with UNS FDG + condition) of ~30%. A massive depletion of the FDG‐cell population upon separation (SEP condition compared with UNS FDG‐condition) was also detected, as expected (Figure S1). Altogether these data demonstrate that the iodixanol gradient‐based separation method performs with the same fidelity when applied to cell populations containing low or high amounts of senescent cells.

## 4 DISCUSSION

Here, we describe for the first time an iodixanol centrifugation‐based method to separate and enrich senescent cells from DXR‐treated HCC cell populations. These separated senescent cells can be efficiently re‐cultured for downstream applications, such as immunofluorescence or immunoblotting. Our gradient centrifugation method is based on OptiPrep, a 60% (w/v) solution of iodixanol in water. OptiPrep solutions have low viscosity and osmolarity and form gradients spontaneously over time, unlike sucrose density solutions.^18^ Solutions based on iodixanol are increasingly appreciated as being superior for cellular imaging^22^ and clinical applications, such as the enrichment of high quality and viable human pancreatic islets destined for transplantation.^23^ Furthermore, previous reports have shown that discontinuous iodixanol gradients (ranging from 1.077 to 1.107 g/mL), upon 1000x *g* centrifugation for 30 minutes, can separate old from young human erythrocytes and sickle erythrocytes^24^ and obtain a reticulocyte‐enriched fraction from whole blood.^25^

Cellular senescence is a basic and instrumental process in evolved organisms that is characterized by cell‐cycle withdrawal.^1^, ^2^, ^3^, ^4^ Cellular senescence typically depends either on: (a) reaching the maximum number of times a cell can divide (the Hayflick limit), which describes replicative senescence; (b) the complex activation of oncogenes, such as RB or p53, which describe oncogene‐induced senescence^26^ or (c) the exposure of cells to agents that induce significant DNA damage.^27^ DXR is a widely used chemotherapeutic and the most prominent member of anthracycline family^2^; it has been used as an anti‐cancer drug for almost 40 years, including for HCC, where it is used as either a monotherapy or a combinatorial therapy.^28^ The mechanisms of DXR‐induced DNA damage include intercalation into DNA, generation of free radicals, DNA cross‐linking, initiation of DNA damage via inhibition of topoisomerase II, eviction of histones and increases in ceramide content.^29^, ^30^, ^31^ These processes occur independently of epigenetic ageing^5^ and are a direct cause of cellular senescence in 40% of DXR‐treated HCC cells.^9^, ^32^, ^33^ DXR triggers different signalling pathways in normal and cancer cells, and is thus responsible for undesired off‐target effects, such as cardiotoxicity.^34^, ^35^

In therapeutic settings, the lack of efficient senescence‐inducing agents and incomplete biological data on the tumour response require a thorough understanding of the biology of chemotherapy‐induced senescent cells, which in turn depends on their isolation from a mixed population comprising adjacent senescent and non‐senescent chemotherapy‐treated normal and cancer cells. Recent high‐throughput approaches to isolate senescent cells from tissues or body fluids, based on their increased cell size, appear more scalable, robust and specific than the ones based on markers that are specific to senescent cells.^6^, ^7^, ^10^, ^11^, ^12^, ^13^ Our optimized iodixanol gradient‐based centrifugation protocol to isolate DXR‐induced senescent HCC cells is simple, quick (~1 hour) and robust. Our protocol is also unique because, unlike previous approaches, it permits cells to be re‐plated on collagen‐coated flasks, despite their low or null proliferative capacity. Using our method, we demonstrate that iodixanol density gradient‐based can greatly enrich for DXR‐induced senescent HepG2 and Huh‐7 HCC cells, characterized by their SA‐β‐galactosidase activity, senescence marker expression (p16, p21, p53, DPP4), proliferation arrest and DNA damage‐dependent γH2A.X foci formation. Interestingly, the appearance and enrichment of DXR‐induced senescence markers were more prominent in HepG2 cells compared with Huh‐7 cells. Huh‐7 cells are more aggressive than HepG2 cells, in vitro and in vivo, and the two cell lines have a different genetic make‐up.^9^, ^19^, ^36^, ^37^ While HepG2 cells express wild‐type p53, Huh‐7 cells express a dominant‐negative mutant form of p53 (Y220C),^38^ which may be involved in resistance to DXR‐induced p53‐mediated cell senescence.^39^

An increased number of senescent cells are associated not only to chemotherapeutic treatment but also to age‐related tissue degeneration towards HCC.^9^, ^13^, ^14^, ^15^ In mice, impaired immune surveillance of senescent hepatocytes induces HCC, indicating that senescence surveillance is important for tumour suppression in the liver.^40^, ^41^ The generation of ‘pure’ cultures of senescent and non‐senescent cells from HCC and non‐tumoural liver tissues might allow future studies to dissect the molecular mechanisms of immune‐mediated clearance, which is triggered by ill‐defined patterns of chemokines and cytokines secreted by different cell populations. More broadly, isolated senescent cells could potentially be used for drug screening and clinical applications in controlled cultured conditions, which will improve our understanding of cancer, cancer treatment and ageing‐related diseases.

## CONFLICT OF INTERESTS

None of the authors declare a conflict of interest.

## Data Availability

The data that support the findings of this study are available from the corresponding author upon reasonable request.
